## [Reviewer comments · Life Science Alliance]

Life Science Alliance

Novel Variants in TECRL Leading to Catecholaminergic Polymorphic Ventricular Tachycardia

Douglas Jones, Jacob Hartung, Elizabeth Lasalle, Alejandro Borquez, Viridiana Murillo, Lucia Guidugli, Kiely James, Stephen Kingsmore, and Nicole Coufal

DOI: <https://doi.org/10.26508/lsa.202402572>

Corresponding author(s): Nicole Coufal, University of California, San Diego

Review Timeline:

Submission Date:	2024-01-04
Editorial Decision:	2024-02-16
Revision Received:	2024-05-06
Editorial Decision:	2024-05-09
Revision Received:	2024-05-09
Accepted:	2024-05-13

Transaction Report:

February 16, 2024

Re: Life Science Alliance manuscript #LSA-2024-02572-T

Nicole Coufal
University of California, San Diego

Dear Dr. Coufal,

Thank you for submitting your manuscript entitled "Rapid Whole Genome Sequencing Identifies Novel Variants in TECRL Leading to Catecholaminergic Polymorphic Ventricular Tachycardia" to Life Science Alliance. The manuscript was assessed by expert reviewers, whose comments are appended to this letter. We invite you to submit a revised manuscript addressing the Reviewer comments.

Thank you for this interesting contribution to Life Science Alliance. We are looking forward to receiving your revised manuscript.

Sincerely,

B. MANUSCRIPT ORGANIZATION AND FORMATTING:

Reviewer #1 (Comments to the Authors (Required)):

Summary: This is a case report of a 12-year-old male who suffered a cardiac arrest followed by successful resuscitation. Rapid whole-genome sequencing (WGS) identified a maternally inherited variant in *TECRL* and an additional large duplication in the same gene. The *TECRL* gene has been implicated in catecholaminergic polymorphic ventricular tachycardia (CPVT) and associated with clinical features of both long QT syndrome (LQTS) and CPVT. Given that *TECRL*-associated CPVT is quite rare, this work is very important to share new variants and phenotypes associated with *TECRL*.

Manuscript:

1. Case Presentation:

- a. Do you have any information about whether the patient was previously running around or swimming before he got knocked over by the waves? It is important to get an idea of the circumstances and adrenergic state preceding his collapse.
- b. I would be hesitant to call Figure 1A TdP. TdP should only be diagnosed based on abnormal repolarization. This is a disorganized rhythm that looks more like VF at times, and one might say polymorphic VT.
- c. Keep consistent with how you reference figures. "Figure 1" vs. "Fig. 1A".
- d. Fig 1B should be referenced in the next sentence: "At that time, an ECG was concerning for worsening prolonged QTc (QTc 568ms, ref <440ms) as well as ST segment prolongation."
- e. "Arrhythmia" is spelled wrong in the third paragraph. Add "(ECG)" after mentioning electrocardiogram. Keep consistent with using HD as an abbreviation.

2. Technical Analysis and Methods:

- a. The abbreviation "rWGS" is inconsistent with what is mentioned in the abstract. Is it rapid whole-genome sequencing or rapid diagnostic genome sequencing?
- b. Variant "calling calling" is repeated.
- c. It is important to spell out abbreviations before they are first used. For instance, CNV and SNV.

3. Variant Classification:

- a. If you mention a novel variant, please report allele frequency or report that it is absent in gnomAD. It would be helpful to report if there are other variants in the same genomic location previously reported.
- b. Mutation causing a change in protein length owing to a termination employs PM4 in ACMG criteria. Making these statements in the variant classification section is necessary, or else the reader does not understand how a likely pathogenic classification was made.
- c. Genes should be italicized in all sections.
- d. The following is a bold statement and should be revised considering that CPVT type 3 is most often inherited in an autosomal recessive pattern. It would also be more fitted for the discussion: "The combination of a heterozygous likely pathogenic nonsense variant and likely pathogenic duplication in the *TECRL* gene would explain the patient's clinical phenotype if the alterations were inherited in trans in the patient."

4. Discussion:

- a. Rephrase: "Neither of these syndromes, however, is completely defined with many genetic etiology not yet established."
- b. LTQS is spelt wrong
- c. There are two important articles on novel variants in *TECRL* that have been recently published that are not reported here. They should be discussed and cited.
-[https://www.heartrhythmcasereports.com/article/S2214-0271\(22\)00255-X/fulltext](https://www.heartrhythmcasereports.com/article/S2214-0271(22)00255-X/fulltext)
-<https://onlinelibrary.wiley.com/doi/full/10.1111/jce.16011>
- d. Cite this statement: "However there remains an increased risk of a cardiac event in patients on beta-blockers, with one long-term follow-up study of 101 patients with CPVT demonstrating an 8-year cardiac event rate of 27% for patients on beta-blockers and potentially fatal arrhythmic events in 11% of CPVT patients."
- e. There are more recent studies that should be cited when mentioning the efficacy of flecainide, in addition to the study by Kannankeril et al which you have already cited).
-<https://pubmed.ncbi.nlm.nih.gov/37886885/>
- f. Patients that are not well managed or experience breakthrough cardiac events on beta-blocker are not typically treated with verapamil, especially in pediatric CPVT. Managing dosing and additional therapy of flecainide is often the next step, not verapamil.
- g. Overall, the discussion needs improvement. It tends to reiterate what is already known about CPVT in general, rather than focusing on the specific phenotype observed in the patient or discussing phenotypes associated with *TECRL* from other published studies.

5. Conclusion:

a. You mention extending the clinical features associated with TECRL to include apical hypertrabeculations. However, you do not discuss this in your discussion. This is a major point. It is important to investigate if other cases of TECRL-associated CPVT have reported any abnormalities on ECHO or MRI.

6. Other:

- a. Keep consistent with capitalization of titles for tables
- b. Arrhythmia spelt wrong in Keywords

Final comments:

The paper requires major revisions, and there are a lot of typos and grammar issues that should be addressed and resolved before resubmission. However, the case report provides novel genetic findings worthy of publication.

Reviewer #2 (Comments to the Authors (Required)):

This case report describes two new pathogenic (likely pathogenic) variants in TECRL gene in a 12 year old boy with CPVT3. It's a well described manuscript. I have few minor suggestions.

1) A reference is recommended in variant classification for CPVT type 3.

2) Discussion section (first paragraph) (please add or rephrase): CPVT 1 and CPVT 2 is associated with RYR2 and CASQ2 pathogenic variants respectively (references: ?). CPVT3 was later described by Bhuiyan et al (2007) (reference: J Cardiovasc. Electrophysiol. 2007;18:1060-6), later the same group elucidated its molecular etiology (Devalla et al. 2016)

2) Discussion. 1st paragraph: Devalla et al described

3) Discussion. 2nd paragraph: CPVT (not CVPT)

Reviewer #3 (Comments to the Authors (Required)):

Submitted paper: Rapid Whole Genome Sequencing Identifies Novel Variants in TERCL Leading to Catecholaminergic Polymorphic Ventricular Tachycardia

Dr Jones and colleagues reported a family harboring rare TERCL variants, in which a young male index patient with history of near SCD was found to carry compound heterozygous variants: c.915T>G [p.Tyr305Ter and chr4:65165944-65185287, dup [4q13.1]. The former is a nonsense variant which can definitely cause LOF of TERCL if it were in a homozygous condition. But, in mother and the proband, the variant was in a heterozygous condition. In the symptomatic proband, an additional 19K duplication encompassing between exon 4 and intron 4 was identified and may cause pathological effects probably through the LOF effects.

There are three different types of inherited catecholamine induced polymorphic ventricular tachycardias also known as CPVTs (CPVT1, CPVT2 and CPVT3). Genes are different, but all may lead to leakage of calcium ions from sarcoplasmic reticulum during diastole. Though still very rare, multiple groups have reported several TECRL mutations causing the CPVT3 phenotype (CPVT3). So, the reviewer wonders if the authors examined both RYR2 (CPVT1) and CASQ2 (CPVT2). Please describe the results of these genetic analyses. In general, in case of CPVT1, RYR2 GOF (gain of function) mutations may lead to the leakage of calcium ions. In contrast, LOF (loss of function) mutations may cause a form of CRDS (Ca ion release deficiency syndrome).

First of all, please paginate your manuscript, otherwise it is difficult to make a communication using page/line numbers.

The reviewer is afraid if this is a clinically real CPVT patient or not. Did the proband have no past history of syncope or any other symptoms suspecting CPVT? Did you perform the exercise stress test? If so, how was result? As far as you diagnose the patient as CPVT, it is obligatory to present the result of exercise stress test. Were there exercise-induced VA, especially bidirectional VT? According to the 2013 Consensus Statement on the Diagnosis and Management of CPVT (Priori et al.), CPVT can be clinically diagnosed in the presence of a structurally normal heart, normal rest ECG, and exercise or catecholamine-induced bidirectional VT and/or polymorphic ventricular premature beats and/or VT in an individual <40 years of age. Otherwise, the discussion on the therapeutic strategy and availability of ICD and/or LCSD would be meaningless, because it is unknown if patient is indeed a CPVT patient or not. The presence of QT prolongation is also unlikely as CPVT, and rather excluding its possibility. The reviewer does not know how the TERCL LOF variants affect cardiac cellular Ca handling, the reviewer guess that the proband was clinically mimicking CRDS, if the exercise stress test was negative.

Minor

The authors stated that the MLPA kit was used for the detection of TERCL variants. The kit was commercially available or custom made? Please give a name of product in the text.

Due to hypotension and depressed cardiac function you mentioned that you used epinephrine and norepinephrine? These agents did not induce polymorphic VAs?

Table 2:

HP:0001663 Ventricular fibrillation section showed that there was No VF, but apparently Fig.1 A presented VF not TdP. Why do you think it is TdP?

Reviewer Comments

We thank the reviewers for their helpful feedback on our manuscript. Please find our responses below in blue.

Reviewer #1 (Comments to the Authors (Required)):

Summary: This is a case report of a 12-year-old male who suffered a cardiac arrest followed by successful resuscitation. Rapid whole-genome sequencing (WGS) identified a maternally inherited variant in TECRL and an additional large duplication in the same gene. The TECRL gene has been implicated in catecholaminergic polymorphic ventricular tachycardia (CPVT) and associated with clinical features of both long QT syndrome (LQTS) and CPVT. Given that TECRL-associated CPVT is quite rare, this work is very important to share new variants and phenotypes associated with TECRL.

Manuscript:

1. Case Presentation:

a. Do you have any information about whether the patient was previously running around or swimming before he got knocked over by the waves? It is important to get an idea of the circumstances and adrenergic state preceding his collapse.

The mother was watching the patient in this case prior to arrest and reported that he was "body-surfing" prior to the incident. This involves falling or jumping into waves and riding their momentum towards shore. He was on vacation so perhaps in an adrenergic state, which we have included in the case report. Otherwise, unfortunately, his adrenergic state is difficult to ascertain.

b. I would be hesitant to call Figure 1A TdP. TdP should only be diagnosed based on abnormal repolarization. This is a disorganized rhythm that looks more like VF at times, and one might say polymorphic VT.

The rhythm displayed is of an unusual polymorphic ventricular tachycardia and has been edited to reflect this terminology.

c. Keep consistent with how you reference figures. "Figure 1" vs. "Fig. 1A".

We have edited the manuscript as requested.

d. Fig 1B should be referenced in the next sentence: "At that time, an ECG was concerning for worsening prolonged QTc (QTc 568ms, ref <440ms) as well as ST segment prolongation."

We have edited the manuscript as requested.

e. "Arrhythmia" is spelled wrong in the third paragraph. Add "(ECG)" after mentioning electrocardiogram. Keep consistent with using HD as an abbreviation.

We have edited the manuscript as requested.

2. Technical Analysis and Methods:

a. The abbreviation "rWGS" is inconsistent with what is mentioned in the abstract. Is it rapid whole-genome sequencing or rapid diagnostic genome sequencing?

This abbreviation refers to rapid whole genome sequencing, we have clarified the text as requested and apologize for the confusion.

b. Variant "calling calling" is repeated.

Addressed, and we apologize.

c. It is important to spell out abbreviations before they are first used. For instance, CNV and SNV.

We have defined these terms as requested.

3. Variant Classification:

a. a. If you mention a novel variant, please report allele frequency or report that it is absent in gnomAD. It would be helpful to report if there are other variants in the same genomic location previously reported.

Both are absent from gnomAD, this was mentioned in the discussion but is now moved to the variant classification section as well.

b. Mutation causing a change in protein length owing to a termination employs PM4 in ACMG criteria. Making these statements in the variant classification section is necessary, or else the reader does not understand how a likely pathogenic classification was made.

We have included this important point as requested in the variant interpretation section.

c. Genes should be italicized in all sections.

Edited as requested.

d. The following is a bold statement and should be revised considering that CPVT type 3 is most often inherited in an autosomal recessive pattern. It would also be more fitted for the discussion: "The combination of a heterozygous likely pathogenic nonsense variant and likely pathogenic duplication in the TECRL gene would explain the patient's clinical phenotype if the alterations were inherited in trans in the patient."

We apologize for any confusion, this is likely an autosomal recessive inheritance, but given that the paternal sample was unavailable, we stated this as being inherited in trans (one from each parent) to avoid the stronger statement of autosomal recessive inheritance. We have clarified.

4. Discussion:

a. Rephrase: "Neither of these syndromes, however, is completely defined with many genetic etiology not yet established."

We have clarified as requested.

b. LTQS is spelt wrong

Corrected as requested, thank you for catching this error.

c. There are two important articles on novel variants in *TECRL* that have been recently published that are not reported here. They should be discussed and cited.

[-https://www.heartrhythmcasereports.com/article/S2214-0271\(22\)00255-X/fulltext](https://www.heartrhythmcasereports.com/article/S2214-0271(22)00255-X/fulltext)

[-https://onlinelibrary.wiley.com/doi/full/10.1111/jce.16011](https://onlinelibrary.wiley.com/doi/full/10.1111/jce.16011)

We have edited the text to include these two references. Specifically adding to the discussion:

Two recent case reports describe similar pathogenic variants in the *TECRL* gene leading to an eventual diagnosis of CPVT. First, Ebrahim and colleagues in 2023 report a case of an 11-year-old boy with cardiac arrest, subsequent prolonged QTc after return of spontaneous circulation, and inducible unstable polymorphic VT with exercise in the electrophysiology lab. A whole-exome sequencing (WES) was notable for a previously described homozygous *TECRL* splice-site variant c.331+1G>A (Ebrahim et al 2023). Secondly, two affected Lebanese siblings experienced exercise induced symptoms with an ECG notable for abnormal QT prolongation, abnormal T-wave morphology and bidirectional ventricular ectopic beats during an exercise stress test. Genetic sequencing confirmed a novel *TECRL* variant, a homozygous c.742_758 del variant that resulted in a protein variant (p.Arg248Cysfs*). In both cases, there was a strong family history of consanguinity and both patients eventually required dual therapy with both a beta-blocker and flecainide (Charafeddine et al 2023).

d. Cite this statement: "However there remains an increased risk of a cardiac event in patients on beta-blockers, with one long-term follow-up study of 101 patients with CPVT demonstrating an 8-year cardiac event rate of 27% for patients on beta-blockers and potentially fatal arrhythmic events in 11% of CPVT patients."

We have added this citation as requested (Hayashi et al 2009).

e. There are more recent studies that should be cited when mentioning the efficacy of flecainide, in addition to the study by Kannankeril et al which you have already cited).

[-https://pubmed.ncbi.nlm.nih.gov/37886885/](https://pubmed.ncbi.nlm.nih.gov/37886885/)

We have cited this as requested.

f. Patients that are not well managed or experience breakthrough cardiac events on beta-blocker are not typically treated with verapamil, especially in pediatric CPVT. Managing dosing and additional therapy of flecainide is often the next step, not verapamil.

We have removed the discussion of verapamil and have focused on flecainide as a mainstay in treatment.

g. Overall, the discussion needs improvement. It tends to reiterate what is already known about CPVT in general, rather than focusing on the specific phenotype observed in the patient or discussing phenotypes associated with TECRL from other published studies.

We have extended the discussion to include other cases of *TECRL* associated arrhythmias as requested.

5. Conclusion:

a. You mention extending the clinical features associated with TECRL to include apical hypertrabeculations. However, you do not discuss this in your discussion. This is a major point. It is important to investigate if other cases of TECRL-associated CPVT have reported any abnormalities on ECHO or MRI.

We have included apical trabeculations in the discussion as well as the conclusions. We have indicated that structural findings have not previously been identified with TECRL variants underscoring the novelty of this case.

6. Other:

a. Keep consistent with capitalization of titles for tables

Corrected as requested.

b. Arrhythmia spelt wrong in Keywords

Corrected.

Final comments:

The paper requires major revisions, and there are a lot of typos and grammar issues that should be addressed and resolved before resubmission. However, the case report provides novel genetic findings worthy of publication.

We thank the reviewer for their careful editing and suggestions and hope we have adequately addressed the comments.

Reviewer #2 (Comments to the Authors (Required)):

This case report describes two new pathogenic (likely pathogenic) variants in TECRL gene in a 12 year old boy with CPVT3. It's a well described manuscript. I have few minor suggestions.

1) A reference is recommended in variant classification for CPVT type 3.

Included as requested.

2) Discussion section (first paragraph) (please add or rephrase): CPVT 1 and CPVT 2 is associated with RYR2 and CASQ2 pathogenic variants respectively (references: ?). CPVT3 was later described by Bhuiyan et al (2007) (reference: J Cardiovasc. Electrophysiol. 2007;18:1060-6), later the same group elucidated its molecular etiology (Devalla et al. 2016)

?We have clarified CVPT 1 and 2 within the discussion as arising from CASQ2 and RYR2 pathogenic variants.

2) Discussion. 1st paragraph: Devalla et al described

Corrected thank you for catching this error.

3) Discussion. 2nd paragraph: CPVT (not CVPT)

Corrected.

Reviewer #3 (Comments to the Authors (Required)):

Submitted paper: Rapid Whole Genome Sequencing Identifies Novel Variants in TERCL Leading to Catecholaminergic Polymorphic Ventricular Tachycardia

Dr Jones and colleagues reported a family harboring rare TERCL variants, in which a young male index patient with history of near SCD was found to carry compound heterozygous variants: c.915T>G [p.Tyr305Ter and chr4:65165944-65185287, dup [4q13.1]. The former is a nonsense variant which can definitely cause LOF of TERCL if it were in a homozygous condition. But, in mother and the proband, the variant was in a heterozygous condition. In the symptomatic proband, an additional 19K duplication encompassing between exon 4 and intron 4 was identified and may cause pathological effects probably through the LOF effects.

There are three different types of inherited catecholamine induced polymorphic ventricular tachycardias also known as CPVTs (CPVT1, CPVT2 and CPVT3). Genes are different, but all may lead to leakage of calcium ions from sarcoplasmic reticulum during diastole. Though still very rare, multiple groups have reported several TERCL mutations causing the CPVT3 phenotype (CPVT3). So, the reviewer wonders if the authors examined both RYR2 (CPVT1) and CASQ2 (CPVT2). Please describe the results of these genetic analyses. In general, in case of CPVT1, RYR2 GOF (gain of function) mutations may lead to the leakage of calcium ions. In contrast, LOF (loss of function) mutations may cause a form of CRDS (Ca ion release deficiency syndrome).

Both RYR2 and CASQ2 were examined as part of the whole genome sequencing analysis and were not found to contain pathogenic mutations.

First of all, please paginate your manuscript, otherwise it is difficult to make a communication using page/line numbers.

We have paginated as requested.

The reviewer is afraid if this is a clinically real CPVT patient or not. Did the proband have no past history of syncope or any other symptoms suspecting CPVT? Did you perform the exercise stress test? If so, how was result? As far as you diagnose the patient as CPVT, it is obligatory to present the result of exercise stress test. Were there exercise-induced VA, especially bidirectional VT? According to the 2013 Consensus Statement on the Diagnosis and Management of CPVT (Priori et al.), CPVT can be clinically diagnosed in the presence of a structurally normal heart, normal rest ECG, and exercise or catecholamine-induced bidirectional VT and/or polymorphic ventricular premature beats and/or VT in an individual <40 years of age. Otherwise, the discussion on the therapeutic strategy and availability of ICD and/or LCSD would be meaningless, because it is unknown if patient is indeed a CPVT patient or not. The presence of QT prolongation is also unlikely as CPVT, and rather excluding its possibility. The reviewer does not know how the TERCL LOF variants affect cardiac cellular Ca handling, the reviewer guess that the proband was clinically mimicking CRDS, if the exercise stress test was negative.

This patient straddles several different phenotypes based on the initial presentation, which is one important reason why this is an interesting case report. It is important to clarify this is not a typical CPVT patient and we have edited the text accordingly. As stated, the proband did not have a history of syncope. This patient was diagnosed with a CPVT phenotype based on the presenting rhythm with unusual features and the likely pathogenic genotype. A stress test was not performed given the recent critical illness. The patient was discharged with a LINQ recorder to continue care in his home state. The guidelines referred to be the reviewer (Priori et al) are adult guidelines and the workup in children is significantly more heterogenous with a relative dearth of exercise stress tests.

Minor

The authors stated that the MLPA kit was used for the detection of TERCL variants. The kit was commercially available or custom made? Please give a name of product in the text.

The rapid whole genome sequencing (rWGS) test is a commercially available test obtained through the Rady's Institute for Genomic Medicine (RCIGM) and has been delineated as such in the text.

Due to hypotension and depressed cardiac function you mentioned that you used epinephrine and norepinephrine? These agents did not induce polymorphic Vas?

The primary agent utilized was norepinephrine although epinephrine was also utilized initially also at low doses. These did not induce polymorphic ventricular arrhythmias at low doses but did exhibit ventricular ectopy which resolved with esmolol, and has been clarified in the text.

Table 2:

HP:0001663 Ventricular fibrillation section showed that there was No VF, but apparently Fig.1 A presented VF not TdP. Why do you think it is TdP?

The patient received a total of three cardioversion shocks in the field over several minutes. During that time, in addition to the potential VT or TdP rhythm displayed in Fig. 1A, some portions of the on-site ECG recorded by the cardioversion device appeared to be VF. We therefore included all these HPO terms in the genome analysis for a comprehensive search.

May 9, 2024

RE: Life Science Alliance Manuscript #LSA-2024-02572-TR

Dr. Nicole G Coufal
University of California, San Diego
Pediatrics
Sanford Consortium
2880 Torrey Pines Scenic Drive Rm 2003
La Jolla, CA 92037

Dear Dr. Coufal,

Thank you for submitting your revised manuscript entitled "Novel Variants in TECRL Leading to Catecholaminergic Polymorphic Ventricular Tachycardia". We would be happy to publish your paper in Life Science Alliance pending final revisions necessary to meet our formatting guidelines.

- please be sure that the authorship listing and order is correct
- please remove the figure from the manuscript file and upload it as a separate file
- please note that the titles in the system and manuscript file must match, the current title entered in the system would do better as the Running Title
- please consult our manuscript preparation guidelines <https://www.life-science-alliance.org/manuscript-prep> and make sure your manuscript sections are in the correct order
- please add your main and table legends to the main manuscript text after the References section
- please provide your Tables in editable .doc or excel format. They can be included at the bottom of the main manuscript file or sent as separate files.
- please be sure that all authors are mentioned in the Author Contributions section in the manuscript text
- please add a callout for Table 1 to your main manuscript text
- please remove the Summary section
- please rename the "Additional Information" section as "Ethics Statement"
- please move the ClinVar accession info into a new section entitled "Data Availability"

LSA now encourages authors to provide a 30-60 second video where the study is briefly explained. We will use these videos on social media to promote the published paper and the presenting author (for examples, see <https://docs.google.com/document/d/1-UWcfbE4pGcDdcgzcmiuJI2XMBJnxKYeqRvLLrLSo8s/edit?usp=sharing>). Corresponding or first-authors are welcome to submit the video. Please submit only one video per manuscript. The video can be emailed to contact@life-science-alliance.org

A. FINAL FILES:

B. MANUSCRIPT ORGANIZATION AND FORMATTING:

Sincerely,

May 13, 2024

RE: Life Science Alliance Manuscript #LSA-2024-02572-TRR

Dr. Nicole G Coufal
University of California, San Diego
Pediatrics
Sanford Consortium
2880 Torrey Pines Scenic Drive Rm 2003
La Jolla, CA 92037

Dear Dr. Coufal,

Thank you for submitting your Research Article entitled "Novel Variants in TECRL Leading to Catecholaminergic Polymorphic Ventricular Tachycardia". It is a pleasure to let you know that your manuscript is now accepted for publication in Life Science Alliance. Congratulations on this interesting work.

DISTRIBUTION OF MATERIALS:

Again, congratulations on a very nice paper. I hope you found the review process to be constructive and are pleased with how the manuscript was handled editorially. We look forward to future exciting submissions from your lab.

Sincerely,
